# Cardiac arrest caused by rapidly increasing ascites in a patient with TAFRO syndrome: a case report

**DOI:** 10.1002/ams2.278

**Published:** 2017-04-17

**Authors:** Masatoshi Okumura, Atsushi Ujiro, Yasunori Otsuka, Hiroshi Yamamoto, Sho Wada, Hirofumi Iwata, Toshiaki Kan, Seiji Miyauchi, Atsushi Hashimoto, Yuko Sato, Yoshihito Fujita, Yoshihiro Fujiwara, Hideki Shimaoka

**Affiliations:** ^1^ Department of Anesthesiology Aichi Medical University Aichi Japan; ^2^ Department of Intensive Care Medicine Osaka‐City General Hospital Osaka Japan

**Keywords:** Ascites, giant lymph node hyperplasia, heart arrest, intra‐abdominal hypertension

## Abstract

**Case:**

Thrombocytopenia, anasarca, fever, renal insufficiency, and organomegaly (TAFRO) syndrome is a newly defined systemic inflammatory disorder with gradual progression of symptoms. A 59‐year‐old man with fever and ascites of unknown cause developed sudden‐onset shock and respiratory failure in the general ward. Cardiac arrest immediately followed. Although he was resuscitated, frequent administration of adrenaline was required to maintain his blood pressure. His circulation was most effectively stabilized by drainage of fluid from his distended abdomen. The volume of discharged ascites reached 4,000 mL at that time, and several liters continued to be discharged for >1 month. The diagnosis of TAFRO syndrome was based on the clinical features and laboratory and histological findings.

**Outcome:**

The ascites volume and concentrations of inflammatory parameters decreased with treatment using several immunosuppressive agents.

**Conclusion:**

The newly defined TAFRO syndrome may be life‐threatening. Patients should be monitored for progression to shock and cardiac arrest, especially those with rapidly increasing ascites.

## Introduction

Thrombocytopenia, anasarca (including pleural fluid and ascites), fever, renal insufficiency, and organomegaly (TAFRO) syndrome is a systemic inflammatory disorder first reported in 2010.^1^, ^2^ This syndrome is considered a variant of multicentric Castleman's disease, but its pathogenetic mechanism remains unknown. Although approximately 30 cases of TAFRO syndrome have been reported, many involved patients on immunosuppressive therapies such as corticosteroids, anti‐interleukin‐6 (anti‐IL‐6) receptor antibody (tocilizumab), and cyclosporin A.^3^, ^4^ The diagnostic criteria, severity classification, and treatment strategy were recently announced by a Japanese group (Table 1).^2^ The mortality rate is 11–12%, and death often occurs after a relatively long disease course.^2^, ^5^ The main causes of death are multiple organ dysfunction unresponsive to immunosuppressive agents and severe infection caused by an immunocompromised state. No reports have described shock and cardiac arrest due to rapidly increasing ascites. We herein report a case involving a patient with TAFRO syndrome that progressed to cardiac arrest due to a large amount of ascites.

**Table 1:** Diagnostic criteria of thrombocytopenia, anasarca, fever, renal insufficiency, and organomegaly (TAFRO) syndrome^2^

All of three major categories and two of four minor categories
Major categories
(1) Anasarca
(2) Thrombocytopenia (platelet count ≤ 100,000/μL)
(3) Systemic inflammation (fever and/or CRP ≥ 2 mg/dL)
Minor categories
(1) Lymph node: Castleman's disease‐like features
(2) Bone marrow: reticulin myelofibrosis and/or increased number of megakaryocytes
(3) Mild organomegaly (hepatomegaly, splenomegaly, and lymphadenopathy)
(4) Progressive renal insufficiency

CRP, C‐reactive protein.

## Case

A 59‐year‐old Japanese man was directly transferred to our hospital with fever, inflammation, ascites, and pleural effusion of unknown cause. He had no medical history other than infection by hepatitis B virus at 24 years of age. He had been well until 3 weeks before admission. One week before admission, he visited a local hospital because of fever (37.8°C) and watery diarrhea. His symptoms did not improve with broad‐spectrum antibiotics. Although laboratory tests, blood cultures, whole‐body computed tomography, echocardiography, and colonoscopy were carried out, the cause of the inflammation remained unknown.

**Figure 1:**
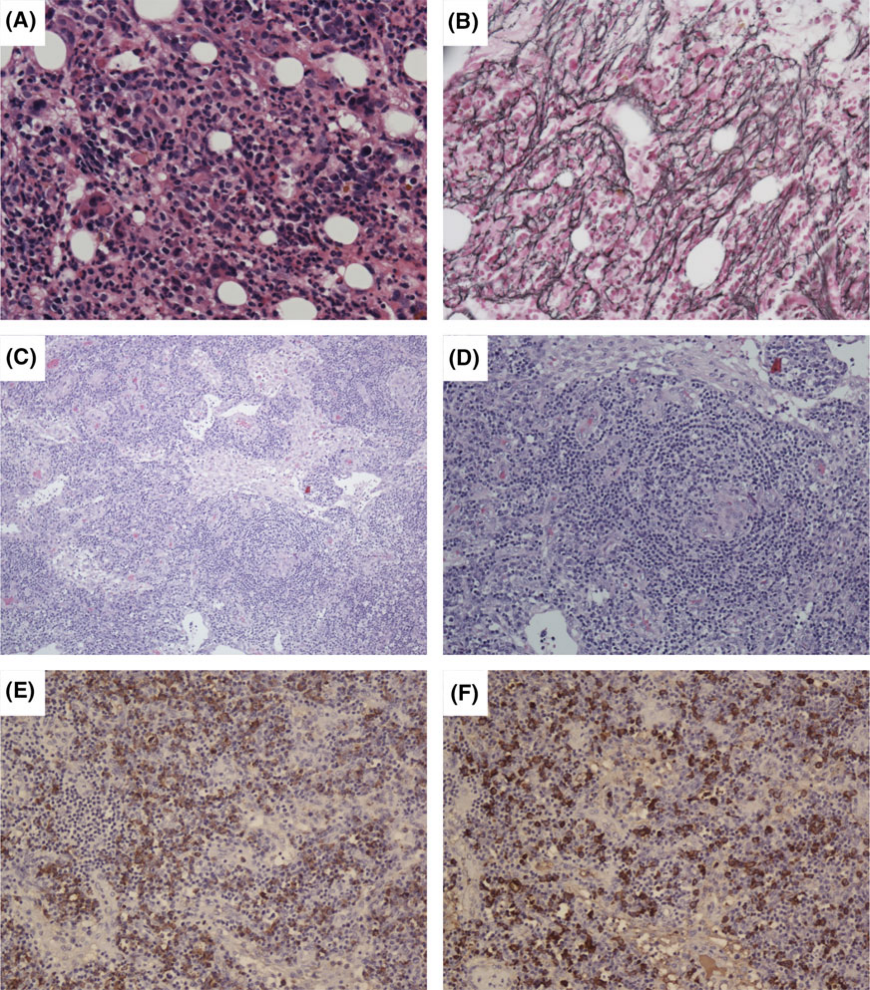
Histological findings of bone marrow (A, B) and lymph node (C–F) in a 59‐year‐old man with thrombocytopenia, anasarca, fever, renal insufficiency, and organomegaly (TAFRO) syndrome. A, C, D, Hematoxylin–eosin staining. B, Silver staining. Kappa (E) and lambda (F) immunostaining.

On admission to our hospital, the patient presented with fever (>38.0°C), abdominal distension, and pitting edema of the legs. All laboratory findings are summarized in Table 2. Laboratory tests showed an increased white blood cell count, mild anemia, thrombocytopenia, hypoalbuminemia, an elevated C‐reactive protein concentration, renal dysfunction, abnormal coagulation, increased serum IL‐6, and increased plasma vascular endothelial cell growth factor. Tests for human herpesvirus 8, HIV, Rickettsia, Aspergillus, and *Mycobacterium tuberculosis* were negative. Immune serology tests and monoclonal immunoglobulin were normal. Tumor markers were negative. Blood and ascites cultures were negative. Computed tomography revealed ascites, bilateral pleural effusion, pericardial effusion, and mild hypertrophy of the bilateral kidneys, but no other enlarged organs or enlarged lymph nodes. Bone marrow biopsy showed increased megakaryocytes and mild reticulin fibrosis (Fig. 1). Right inguinal lymph node biopsy showed lymph follicles with angiosclerosis, atrophic germinal centers, vascular proliferation, and infiltration of plasma cells. Moreover, the lymph node showed no evidence of monoclonal plasma cells. These findings were compatible with mixed‐type Castleman's disease and TAFRO syndrome.

**Table 2:** Laboratory data of a 59‐year‐old man with thrombocytopenia, anasarca, fever, renal insufficiency, and organomegaly (TAFRO) syndrome on admission to our hospital

**Complete blood count**			**Biochemistry**			**Virologic test**		
WBC	19.6	10^3^/μL	TP	5.0	g/dL	HIV	(−)	
Stab	9	%	Albumin	1.5	g/dL	HBs‐Ag	(−)	
Seg	82	%	BUN	40.5	mg/dL	HBs‐Ab	(+)	
Eosi	0	%	Creatinine	1.25	mg/dL	HBc‐Ab	(+)	
Baso	0	%	T‐Bil	1.1	mg/dL	HCV‐Ab	(−)	
Mono	4	%	AST	22	IU/L	HHV‐8‐DNA	(−)	
Lymp	5	%	ALT	12	IU/L	**Immunologic test**		
RBC	4.11	10^6^/μL	LDH	279	IU/L	IgG	1184	mg/dL
Hb	11.6	g/dL	ALP	532	IU/L	IgA	224	mg/dL
Ht	33.5	%	Na	136	mEq/L	IgM	19	mg/dL
MCV	81.5	fl	K	4.2	mEq/L	PAIgG	78.8	ng/10^7^ calls
MCH	28.2	pg	Cl	103	mEq/L	RF	(−)	
MCHC	34.6	%	Ca	7.5	mg/dL	ANA	(−)	
Platelet	5.7	10^4^/μL	CK	25	IU/L	Anti‐SS‐A Ab	(−)	
**Coagulation test**			T‐Chol	68	mg/dL	Anti‐dsDNA Ab	(−)	
PT	52.2	%	CRP	19.2	mg/dL	aCL‐ß2GPI	(−)	
PT‐INR	1.29		Procalcitonin	3.77	ng/mL	PR‐3 ANCA	(−)	
APTT	31.6	s	Ferritin	1149	ng/mL	MPO‐ANCA	(−)	
Fbg	368	mg/dL	IL‐6	14	pg/mL	IgG4	71	mg/dL
FDP	79.4	μg/mL	VEGF	119	pg/mL			
AT	42	%	sIL‐2R	1567	IU/mL			

aCL‐β2GPI, anticardiolipin–β2‐glycoprotein I; ALP, alkaline phosphatase; ALT, alanine aminotransferase; ANA, antinuclear antibody; APTT, activated partial prothrombin time; AST, aspartate aminotransferase; AT, antithrombin; Baso, basophils; BUN, blood urea nitrogen; CK, creatine kinase; CRP, C‐reactive protein; dsDNA, double‐stranded DNA; Eosi, eosinophils; Fbg, fasting blood glucose; FDP, fibrin degradation products; Hb, hemoglobin; HBc‐Ab, hepatitis B core antibody; HBs‐Ab, hepatitis B surface antibody; HBs‐Ag, hepatitis B surface antigen; HCV‐Ab, hepatitis C virus antibody; HHV‐8, human herpesvirus 8; Ht, hematocrit; Ig, immunoglobulin; IL, interleukin; LDH, lactate dehydrogenase; Lymp, lymphocytes; MCH, mean corpuscular hemoglobin; MCHC, mean corpuscular hemoglobin concentration; MCV, mean corpuscular volume; Mono, monocytes; MPO‐ANCA, myeloperoxidase–antineutrophil cytoplasmic antibodies; PAIgG, platelet‐associated immunoglobulin G; PR‐3‐ANCA, proteinase‐3–antineutrophil cytoplasmic antibodies; PT, prothrombin time; PT‐INR, prothrombin time–international normalized ratio; RBC, red blood cells; RF, rheumatoid factor; Seg, segmented neutrophils; sIL‐2R, soluble interleukin‐2 receptor; Stab, stab neutrophils; T‐Bil, total bilirubin; T‐Chol, total cholesterol; TP, total protein; VEGF, vascular endothelial growth factor; WBC, white blood cells.

Reference range: IL‐6, <8 pg/mL; VEGF, <38.3 pg/mL; sIL‐2R, 145–519 IU/mL; IgG4, 4–108 mg/dL.

**Figure 2:**
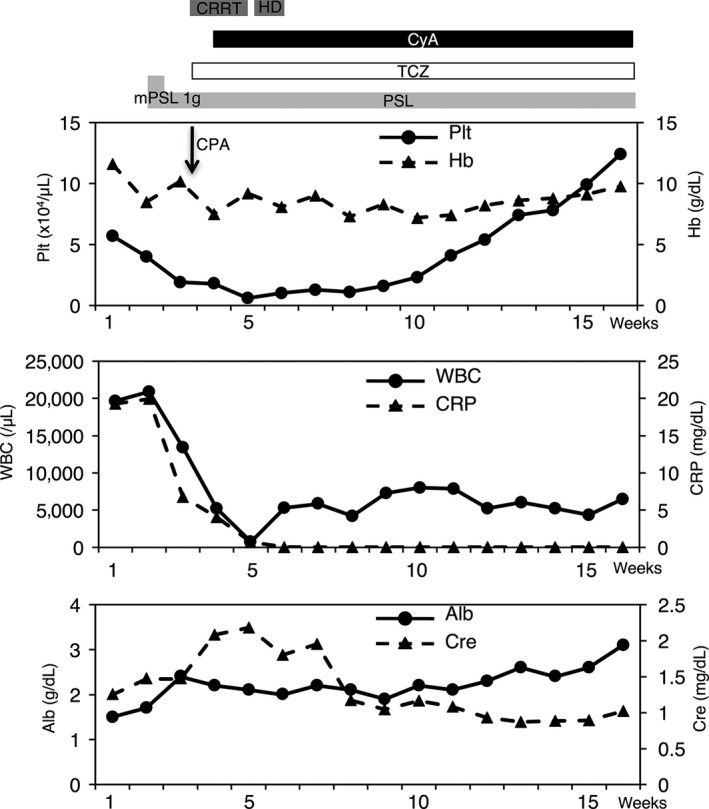
Clinical course from hospitalization to discharge in a 59‐year‐old man with thrombocytopenia, anasarca, fever, renal insufficiency, and organomegaly (TAFRO) syndrome. Alb, albumin; Cre, creatinine; CRP, C‐reactive protein; CRRT, continuous renal replacement therapy; CyA, cyclosporin A; Hb, hemoglobin; HD, hemodialysis; mPSL, methylprednisolone; Plt, platelets; PSL, prednisolone; TCZ, tocilizumab; WBC, white blood cells.

After admission, the fever and anasarca deteriorated despite administration of meropenem (1 g every 8 h), vancomycin (trough level of 15–20 μg/mL), doxycycline (100 mg every 12 h), and diuretics. On day 10, high‐dose methylprednisolone (1 g/day for 3 days) was given, and prednisolone (1 mg/kg per day) was started on day 13 (Fig. 2). On day 17, the patient developed severe, sudden‐onset respiratory distress and circulatory failure in the general ward and went into pulseless electrical activity within 1 min. After resuscitation and transport to the intensive care unit, his vital signs were unstable; his blood pressure was 67/35 mmHg, heart rate was 113 b.p.m., and oxygen saturation was 95% with 10‐L oxygen flow. In the intensive care unit, frequent administration of 0.1 mg adrenaline was necessary to maintain his blood pressure. The most effective intervention to alleviate shock was drainage of fluid from his firm, distended abdomen. We were able to maintain his blood pressure by draining 4,000 mL of ascites. Following drainage, cardiac ultrasonography revealed a change from hypokinetic to normokinetic contraction of the left ventricle. The ascites reached a total of 7,300 mL on the first day, and drainage of an average of 3,576 mL/day of ascites continued for 6 weeks. Frequent treatment with crystalloid solutions, albumin, fresh frozen plasma, and plasma concentrate was required to reduce the circulatory volume, albumin concentration, and coagulation factors.

On day 18, TAFRO syndrome was diagnosed according to the patient's clinical features, laboratory data, and lymph node and bone marrow pathology. Treatment with 8 mg/kg tocilizumab every other week was initiated (Fig. 2). The serum IL‐6 concentration increased further to 567 pg/mL at this point. Some inflammatory parameters improved for 5 days after initiating tocilizumab therapy; however, the anasarca worsened. Therefore, starting on day 24, cyclosporin A (250 mg/day) was given along with prednisolone and tocilizumab. The patient's clinical symptoms and laboratory data gradually improved over 3 months. He was discharged home on day 112.

## Discussion

This case suggests that TAFRO syndrome is associated with acute life‐threatening symptoms and leads to cardiac arrest due to rapidly increasing ascites.

The mortality rate is 11–12%.^2^, ^5^ In some reports, the cause of death was multiple organ failure secondary to deteriorating TAFRO syndrome over a 2‐month period and septic shock associated with febrile neutropenia during an 8‐month observation period.^2^ Although no reports have described acute life‐threatening symptoms in patients with TAFRO syndrome, we assume that TAFRO syndrome may cause death in the acute phase. Some patients with TAFRO syndrome might die before diagnosis because TAFRO syndrome is uncommon and difficult to diagnose. Therefore, special attention should be given to patients with acute deterioration of TAFRO syndrome.

The diagnostic criteria for TAFRO syndrome were announced by two groups.^2^, ^5^ Both these criteria emphasize clinical findings, however, one requires the histological feature in lymph node, the other does not. In the present case, anasarca, thrombocytopenia, systemic inflammation, Castleman's disease‐like feature in lymph node, reticulin myelofibrosis in bone marrow, and renal insufficiency are consistent with the criteria. Lymph node findings partially met the new criteria.^5^ However, according to past cases, there is no contradiction in the pathological findings of TAFRO syndrome. As a result, this case is diagnosed as TAFRO syndrome.

This syndrome leads to cardiac arrest caused by rapidly increasing ascites. Although 96–100% of patients with TAFRO syndrome develop anasarca, the quantity of ascites and incidence of cardiac arrest due to ascites have not been reported.^2^, ^5^ In the present case, 4,000 mL of ascites was drained at the time of cardiac arrest, and 3,000–5,000 mL/day of ascites was continuously drained for 6 weeks despite immunosuppressive therapy. The causes of cardiac arrest include reduced venous return secondary to abdominal compartment syndrome by rapidly increasing ascites as well as impaired cardiac compliance and contractility by elevation of the diaphragm. In addition, hypovolemia due to a large amount of anasarca is a cause of reduced venous return.

The most common causes of abdominal compartment syndrome are abdominal surgery, abdominal trauma, retroperitoneal bleeding after aortic surgery, and burns to the abdominal wall. Only two cases of cardiac arrest due to acute abdominal compartment syndrome have been reported.^6^, ^7^ In both cases, intestinal perforation was the cause of abdominal compartment syndrome and cardiac arrest during colonoscopy, and pulses were detected when the distended abdomen was decompressed using a 14‐gauge needle. Indeed, a pulse was also detected after intra‐abdominal drainage in the present case.

To the best of our knowledge, no reports of cardiac arrest caused by rapidly increasing ascites in patients with TAFRO syndrome have been published. The ascites associated with TAFRO syndrome might increase very rapidly and reach a rate of several liters per day. We should assume that these changes lead to cardiac arrest secondary to abdominal compartment syndrome and hypovolemia. Both volume resuscitation and decompression of the distended abdomen are required as soon as possible in such patients.

## Conflict of Interest

None declared.
